# Supplementary material for: Pre-Clinical Evaluation of Efficacy and Safety of Human Limbus-Derived Stromal/Mesenchymal Stem Cells with and without Alginate Encapsulation for Future Clinical Applications
Source: Cells. 2023 Mar 11;12(6):876. doi: 10.3390/cells12060876 (PMC10047711; doi:10.3390/cells12060876)
Supplement: Supplementary file 1 [file cells-12-00876-s001.zip › cells-2198893-supplementary.pdf]

Pre-clinical evaluation of efficacy and safety of human limbus-derived stromal/mesenchymal stem cells with and without alginate encapsulation for future clinical applications

Mukesh Damala, [1,2], Abhishek Sahoo, [1], Naveen Pakalapati, [1], Vivek Singh, [1,*] and Sayan Basu [1,*]

Supplementary Information

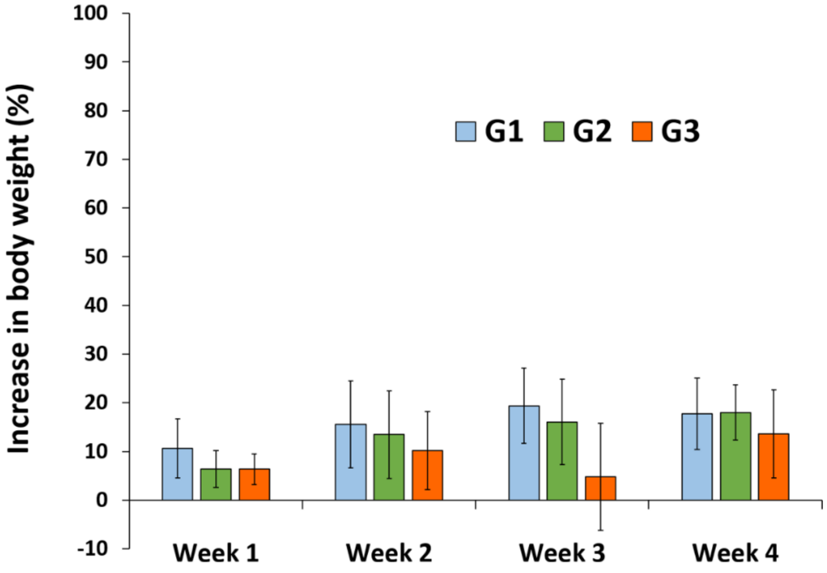

Supplementary Figure S1. The rabbit's relative body weight growth (%) after hLMSC therapy. Post-treatment rabbit weight graph. Three hLMSC-treated rabbit groups (n=6). On treatment day and weeks 1–4, they were weighed in kg. Plotting the weight gain since treatment. Mean ± SD; $p>0.05$. Statistical analysis was done with t-test. G1: Sham-treated group; G2: Cells without encapsulation/ transit (En- hLMSCs); G3: Cells post-encapsulation and transit (En+ hLMSCs).

Supplementary Table S1. Ophthalmic lesion grading.

Observation	Score
**Corneal opacity**	
No ulcers or opacities	0
Scattered or diffuse opacity; iris details clearly visible.	1
Visible translucent area; iris details obscured.	2
Nacreous area; no iris details; pupil barely visible.	3
Opaque cornea covers up iris.	4
**Iris**	
Normal	0
Deepened rugae, congestion, swelling, moderate circumcorneal hyperemia, or injection; light-reactive iris	1
Bleeding, destruction, or light insensitivity (any or all of these)	2
**Conjunctival Redness** (refers to palpebral and bulbar conjunctivae; excluding cornea and iris)	
Normal blood vessels	0
Hyperaemic vessels exist (injected)	1
Diffuse, crimson; vessels hard to see.	2
Diffuse beefy red	3
**Chemosis** (swelling of lids and/or nictitating membranes**)**	
No swelling (Normal)	0
Extra swelling (includes nictitating membranes)	1
Swelling and lids partially everted	2
Swelling, lids half closed	3
Swelling, with lids more than half closed	4

Ophthalmic parameters and severity scores. En-/En+ hLMSCs treated three groups of six rabbits with corneal wounds.

Supplementary Table S2. Summary of ocular lesions in ophthalmic observations.

Time Point	G1 Normal Eye	G1 Treated Eye	G2 Normal Eye	G2 Treated Eye	G3 Normal Eye	G3 Treated Eye
**Conjunctival redness**						
Pre-dose	0 (6 of 6)	0 (6 of 6)	0 (6 of 6)	0 (6 of 6)	0 (6 of 6)	0 (6 of 6)
3 hours	0 (6 of 6)	**1 (6 of 6)**	0 (6 of 6)	**1 (6 of 6)**	0 (6 of 6)	0 (6 of 6)
6 hours	0 (6 of 6)	**1 (1 of 6)**	0 (6 of 6)	**1 (6 of 6)**	0 (6 of 6)	0 (6 of 6)
12 hours	0 (6 of 6)	0 (6 of 6)	0 (6 of 6)	0 (6 of 6)	0 (6 of 6)	0 (6 of 6)
24 hours	0 (6 of 6)	0 (6 of 6)	0 (6 of 6)	0 (6 of 6)	0 (6 of 6)	0 (6 of 6)
day 7	0 (6 of 6)	0 (6 of 6)	0 (6 of 6)	0 (6 of 6)	0 (6 of 6)	0 (6 of 6)
day 14	0 (6 of 6)	0 (6 of 6)	0 (6 of 6)	0 (6 of 6)	0 (6 of 6)	0 (6 of 6)
day 21	0 (6 of 6)	0 (6 of 6)	0 (6 of 6)	0 (6 of 6)	0 (6 of 6)	0 (6 of 6)
day 28	0 (6 of 6)	0 (6 of 6)	0 (6 of 6)	0 (6 of 6)	0 (6 of 6)	0 (6 of 6)
**Corneal opacity**						
Pre-dose	0 (6 of 6)	0 (6 of 6)	0 (6 of 6)	0 (6 of 6)	0 (6 of 6)	0 (6 of 6)
3 hours	0 (6 of 6)	0 (6 of 6)	0 (6 of 6)	0 (6 of 6)	0 (6 of 6)	0 (6 of 6)
6 hours	0 (6 of 6)	0 (6 of 6)	0 (6 of 6)	0 (6 of 6)	0 (6 of 6)	0 (6 of 6)
12 hours	0 (6 of 6)	0 (6 of 6)	0 (6 of 6)	0 (6 of 6)	0 (6 of 6)	0 (6 of 6)
24 hours	0 (6 of 6)	0 (6 of 6)	0 (6 of 6)	0 (6 of 6)	0 (6 of 6)	0 (6 of 6)
day 7	0 (6 of 6)	0 (6 of 6)	0 (6 of 6)	0 (6 of 6)	0 (6 of 6)	0 (6 of 6)
day 14	0 (6 of 6)	0 (6 of 6)	0 (6 of 6)	0 (6 of 6)	0 (6 of 6)	0 (6 of 6)
day 21	0 (6 of 6)	0 (6 of 6)	0 (6 of 6)	0 (6 of 6)	0 (6 of 6)	0 (6 of 6)
day 28	0 (6 of 6)	0 (6 of 6)	0 (6 of 6)	0 (6 of 6)	0 (6 of 6)	0 (6 of 6)
**Lesions in Iris**						
**Pre-dose**	0 (6 of 6)	0 (6 of 6)	0 (6 of 6)	0 (6 of 6)	0 (6 of 6)	0 (6 of 6)
3 hours	0 (6 of 6)	0 (6 of 6)	0 (6 of 6)	0 (6 of 6)	0 (6 of 6)	0 (6 of 6)
6 hours	0 (6 of 6)	0 (6 of 6)	0 (6 of 6)	0 (6 of 6)	0 (6 of 6)	0 (6 of 6)
12 hours	0 (6 of 6)	0 (6 of 6)	0 (6 of 6)	0 (6 of 6)	0 (6 of 6)	0 (6 of 6)
24 hours	0 (6 of 6)	0 (6 of 6)	0 (6 of 6)	0 (6 of 6)	0 (6 of 6)	0 (6 of 6)
day 7	0 (6 of 6)	0 (6 of 6)	0 (6 of 6)	0 (6 of 6)	0 (6 of 6)	0 (6 of 6)
day 14	0 (6 of 6)	0 (6 of 6)	0 (6 of 6)	0 (6 of 6)	0 (6 of 6)	0 (6 of 6)
day 21	0 (6 of 6)	0 (6 of 6)	0 (6 of 6)	0 (6 of 6)	0 (6 of 6)	0 (6 of 6)
day 28	0 (6 of 6)	0 (6 of 6)	0 (6 of 6)	0 (6 of 6)	0 (6 of 6)	0 (6 of 6)
**Aqueous humour**						
Pre-dose	0 (6 of 6)	0 (6 of 6)	0 (6 of 6)	0 (6 of 6)	0 (6 of 6)	0 (6 of 6)
3 hours	0 (6 of 6)	0 (6 of 6)	0 (6 of 6)	0 (6 of 6)	0 (6 of 6)	0 (6 of 6)
6 hours	0 (6 of 6)	0 (6 of 6)	0 (6 of 6)	0 (6 of 6)	0 (6 of 6)	0 (6 of 6)
12 hours	0 (6 of 6)	0 (6 of 6)	0 (6 of 6)	0 (6 of 6)	0 (6 of 6)	0 (6 of 6)
24 hours	0 (6 of 6)	0 (6 of 6)	0 (6 of 6)	0 (6 of 6)	0 (6 of 6)	0 (6 of 6)
day 7	0 (6 of 6)	0 (6 of 6)	0 (6 of 6)	0 (6 of 6)	0 (6 of 6)	0 (6 of 6)
day 14	0 (6 of 6)	0 (6 of 6)	0 (6 of 6)	0 (6 of 6)	0 (6 of 6)	0 (6 of 6)
day 21	0 (6 of 6)	0 (6 of 6)	0 (6 of 6)	0 (6 of 6)	0 (6 of 6)	0 (6 of 6)
day 28	0 (6 of 6)	0 (6 of 6)	0 (6 of 6)	0 (6 of 6)	0 (6 of 6)	0 (6 of 6)

Ophthalmic observation scores according to severity (Table 1) of ocular lesions. G1: Sham-treated; G2: En-hLMSCs; G3: En+ hLMSCs.

Supplementary Table S3. Serial evaluation of IOP after treatment with *En*+/*En*- hLMSCs.

**Intraocular pressure of the treated eyes**									
**Group**	**Pre-dose**	**3 hours**	**6 hours**	**12 hours**	**24 hours**	**Day 7**	**Day 14**	**Day 21**	**Day 28**
G1	10.5	10.0	11.0	11.0	13.0	13.0	12.0	13.0	12.5
G2	12.5	9.0	10.0	11.5	13.0	11.0	12.0	15.0	12.0
G3	12.0	12.0	9.0	11.0	12.5	13.0	13.0	11.0	12.5
p-value	0.185	0.063	0.268	0.855	0.953	0.154	0.718	0.069	0.349
**Intraocular pressure of the normal eyes**									
G1	12	10.5	13	13.5	13.5	14	13	13.5	13
G2	13.5	8	13.5	13	13.5	12.5	12	13	12
G3	12.5	10	12.5	13.5	13.5	12	13.5	12.5	12
p- value	0.715	0.067	0.877	0.792	0.999	0.056	0.586	0.498	*0.017*

Scores of ophthalmic observations based on ocular lesion severity (Table 1). G1—Sham-treated group; G2—En-hLMSCs; G3—En+ hLMSCs. Statistical analysis done with Kruskal Wallis test.

Supplementary Table S4. Serum and tear immunological and inflammatory markers after En+/En- hLMSCs treatment**.**

Group	1 hours	3 hours	6 hours	12 hours	24 hours	Day 7	Day 14	Day 21	Day 28
**Median levels of inflammatory markers in serum**									
**IgE (µg/mL)**									
G1	0.961	NA*	0.684	0.327	0.592	1.205	0.789	0.84	0.626
G2	0.787	NA*	0.659	0.736	0.685	1.555	1.085	1.655	0.537
G3	1.845	NA*	1.588	2.28	2.145	1.875	2.165	1.865	1.95
p-value	*0.048*	NA*	0.067	*0.016*	*0.022*	0.549	*0.024*	0.206	*0.017*
**IL-6 (pg/mL)**									
G1	1025	NA*	294.1	345.2	462.2	162.1	251.8	199.9	363.6
G2	735.5	NA*	291.6	209.6	189.8	148.3	212.5	207.9	211.2
G3	373.6	NA*	209.2	176.3	195.6	180.4	171.2	215.6	174.6
p-value	*0.019*	NA*	0.737	0.504	0.258	0.222	0.331	0.549	0.296
**TNF-α (pg/mL)**									
G1	171.4	NA*	26	44.2	37.1	36.6	31.2	49.5	41.4
G2	165.7	NA*	28.9	23.7	37.3	33.9	25	34.3	27.1
G3	110.9	NA*	18.2	20.1	15.9	26.9	24.1	31.7	23.9
p-value	0.386	NA*	0.199	0.544	0.089	0.354	0.471	0.271	0.187
**Median levels of inflammatory markers in tears**									
**IgE (µg/mL)**									
G1	32.5	29.5	30.2	31.8	25.5	17.77	25.2	9.7	23.2
G2	165.7	98.5	26.4	17.5	27.1	22.73	17.9	18.5	22.1
G3	30.7	26.4	26.1	26.9	21.1	5.87	18.9	7.4	18
p-value	*0.023*	*0.026*	0.471	0.341	0.737	0.116	0.528	0.344	0.603
**IL-6 (pg/mL)**									
G1	284.3	274.5	345.2	325.5	275.7	316.2	345.2	454.9	357.7
G2	177.9	188.7	192.7	154.5	191.8	198.6	166.2	176.3	195.1
G3	282.2	269	257.8	279.7	114.9	98.3	91.4	100.8	110.6
p-value	*0.021*	*0.008*	*0.012*	*0.012*	*0.006*	*0.005*	*0.004*	*0.004*	*0.014*
**TNF-α (pg/mL)**									
G1	67.2	51.9	67.9	71.1	56.4	50.5	59.6	61.1	54.5
G2	28.7	27.6	27.9	30.2	31.9	30.7	31.4	30.8	32.9
G3	31.1	43.1	42.6	47.9	34.7	33.1	27.3	25.6	42.6
p-value	0.091	0.108	*0.005*	*0.004*	*0.253*	*0.109*	*0.071*	*0.071*	*0.028*

Median levels and Kruskal Wallis test analysis (non-parametric one-way ANOVA). *3-hour blood samples were not taken, but tear samples were. G1: Sham-treated; G2: En-hLMSCs; G3: En+ hLMSCs.

Supplementary Table S5. Hematological observations made on rabbits following treatment with hLMSCs.

S. No.	Parameter	G1 (sham)	G2 (*En*- hLMSCs)	G3 (*En*+ hLMSCs)	Units
1.	Haematocrit	44.8 ± 3.31	42.5 ± 4.87	40.73 ± 2.35	%
2.	Hemoglobin	13.83 ± 1.06	13.10 ± 1.61	12.93 ± 0.72	gm/dL
3.	Mean Corpuscular Volume	72.35 ± 2.59	70.33 ± 3.23	70.90 ± 1.97	fL
4.	Platelets	384 ± 152.69	330.5 ± 69.85	461.17 ± 215.31	10^3/μL
5.	Red Blood Corpuscles	6.19 ± 0.4	6.06 ± 0.77	5.75 ± 0.24	10^6/μL
6.	White blood Corpuscles	9.97 ± 3.04	7.93 ± 1.54	8.63 ± 3.01	10^3/μL
7.	Differential Count				
	Neutrophils	40.67 ± 8.90	32.0 ± 5.29	3.83 ±15.68	%
	Lymphocytes	52.50 ± 10.08	62.67 ± 5.76	50.17 ± 15.42	%
	Monocytes	4.5 ± 1.38	3.67 ± 0.75	4.00 ± 0.58	%
	Eosinophil	2.33 ± 0.75	1.67 ± 0.47	2.0 ± 0.58	%
	Basophils	0.00 ± 0.00	0.00 ± 0.00	0.00 ± 0.00	%
8.	Reticulocyte Count	1.83 ± 0.08	1.86 ± 0.06	1.87 ± 0.05	%
9.	Bleeding Time	3.06 ± 0.29	3.25 ± 0.14	3.08 ± 0.30	minutes
10.	Coagulation Time	6.98 ± 0.33	7.22 ± 0.37	6.98 ± 0.33	minutes
11.	Prothrombin Time	16.17 ± 1.07	16.33 ± 0.94	16.67 ± 1.11	seconds
12.	Activated Partial Thromboplastin Time	39.67 ± 4.03	41.00 ± 2.08	40.33 ± 1.70	seconds
13.	Erythrocyte Sedimentation Rate	4.0 ± 3.21	5.67 ± 3.35	5.33 ± 1.89	mm/1st hour

Group G1, G2, and G3 rabbit blood parameters. Test item-administered animals did not differ from vehicle control group animals. $p>0.05$. G1: Sham-treated; G2: En-hLMSCs; G3: En+ hLMSCs. Statistical analysis done with t-test.

Supplementary Table S6. Rabbit clinical chemistry after En-/En+ hLMSC treatment.

S. No.	Parameter	G1 (sham)	G2 (*En*- hLMSCs)	G3 (*En*+ hLMSCs)	Units
1.	Serum Glucose	112.83 ± 17.49	117.67 ± 12.76	115.33 ± 6.39	mg/dL
2.	Blood Urea Nitrogen	21.50 ± 2.99	21.67 ± 3.35	22.50 ± 3.64	mg/dL
3.	Serum Creatinine	0.95 ± 0.13	0.85 ± 0.08	0.98 ± 0.29	mg/dL
4.	Serum Total Bilirubin	0.05 ± 0.00	0.05 ± 0.01	0.05 ± 0.00	mg/dL
5.	Alanine Aminotransferase	61.83 ± 12.56	59.50 ± 26.91	81.67± 32.03	IU/L
6.	Aspartate Aminotransferase	62.17 ± 13.03	74.17 ± 67.73*	61.00 ± 11.65	IU/L
7.	Serum Alkaline phosphatase	86.67 ± 39.77	134.83 ± 48.47	107.83 ± 28.37	IU/L
8.	Serum Total Protein	6.0 ± 0.18	5.63 ± 0.38	5.82 ± 0.25	g/dL
9.	Serum Albumin	2.40 ± 0.07	2.36 ± 0.20	2.46 ± 0.10	g/dL
10.	Globulin	3.60 ± 0.21	3.27 ± 0.21*	3.38 ± 0.23	g/dL
11.	Serum Total Cholesterol	56.33 ± 29.03	59.00 ± 13.76	74.83 ± 37.75	mg/dL
12.	High-density lipoprotein	23.50 ± 11.91	29.17 ± 9.15	30.83 ± 10.35	mg/dL
13.	Low-density lipoprotein	27.00 ± 16.90	25.67 ± 7.36	36.50 ± 23.68	mg/dL
14.	Serum Phosphorous	5.83 ± 0.39	6.22 ± 0.46	7.35 ± 1.11	mg/dL
15.	Serum Calcium	12.98 ± 0.19	12.80 ± 0.46	12.67 ± 0.40	mg/dL
16.	Serum Sodium	158.40 ± 3.19	153.26 ± 5.01*	152.47 ± 1.86	mmol/L
17.	Serum Potassium	4.84 ± 0.21	4.73 ± 0.36	5.50 ± 0.91	mmol/L
18.	Gamma-glutamyl transferase	8.42 ± 3.30	10.65 ± 3.46	9.48 ± 4.33	IU/L

Group G1, G2, and G3 rabbit biochemical parameters. Clinical parameters did not affect systemic organs. *$p \leq 0.05$. G1: Sham-treated; G2: En-hLMSCs; G3: En+ hLMSCs. Statistical analysis was done with t-test.

Supplementary Table S7. Summary of histopathological observations of rabbit organs post-treatment with hLMSCs.

S.No.	Organ	Observations	G1 (sham)	G2 (*En*- hLMSCs)	G3 (*En*+ hLMSCs)
1.	Liver	Sinusoidal hemorrhage	**2/6***	**2/6**	**2/6**
		Inflammation/necrosis/infiltration of inflammatory cells in centrilobular portal region	**1/6**	0/6	0/6
		No Abnormality Detected (NAD)	3/6	4/6	4/6
2.	Spleen	NAD	6/6	6/6	6/6
3.	Lungs	Thickening of alveolar wall/ inflammation	**5/6**	**5/6**	**4/6**
		NAD	1/6	1/6	2/6
4.	Heart	NAD	6/6	6/6	6/6
5.	Aorta	NAD	6/6	6/6	6/6
6.	Kidney	Tubular degeneration	0/6	**1/6**	**1/6**
		Tubular/ interstitial inflammation	**3/6**	**1/6**	**1/6**
		NAD	3/6	4/6	4/6
7.	Adrenals	NAD	6/6	6/6	6/6
8.	Brain	Foci of cerebral necrosis/ perivascular cuffing	**2/6**	**1/6**	0/6
		NAD	4/6	5/6	6/6
9.	Thyroid and Para thyroid	NAD	6/6	6/6	6/6
10.	Trachea	NAD	6/6	6/6	6/6
11.	Oesophagus	NAD	6/6	6/6	6/6
12.	Duodenum	NAD	6/6	6/6	6/6
13.	Jejunum	NAD	6/6	6/6	6/6
14.	Ileum	Sub mucosal lymphoid tissue hyperplasia	**1/6**	**1/6**	0/6
		NAD	5/6	5/6	6/6
15.	Colon	NAD	6/6	6/6	6/6
16.	Rectum	NAD	6/6	6/6	6/6
17.	Lymph nodes	NAD	6/6	6/6	6/6
18.	Pancreas	NAD	6/6	6/6	6/6
19.	Thymus	NAD	6/6	6/6	6/6
20.	Urinary bladder	NAD	6/6	6/6	6/6
21.	Stomach	NAD	6/6	6/6	6/6
22.	Skeletal muscle	NAD	6/6	6/6	6/6
23.	Skin	NAD	6/6	6/6	6/6
24.	Spinal cord	NAD	6/6	6/6	6/6
25.	Middle ear	NAD	6/6	6/6	6/6
26.	Eye	NAD	6/6	6/6	6/6

*Key: No. of animals showing observation/Total no. of animals. Group 1 received no treatment (sham group); Group 2 received En- hLMSCs; and Group 3 received En+ hLMSCs.

Supplementary Table S8. Organ weight summaries after hLMSC treatment in rabbits.

S. No.	Organ	G1 (sham)	G2 (*En*- hLMSCs)	G3 (*En*+ hLMSCs)
1.	Brain	8.42 ± 0.85	8.11 ± 0.63	8.10 ± 0.85
2.	Heart	6.28 ± 0.53	7.57 ± 1.17	7.01 ± 1.62
3.	Liver	73.15 ± 10.09	80.70 ± 16.78	71.72 ± 15.87
4.	Spleen	1.05 ± 0.33	1.14 ± 0.39	0.76 ± 0.20
5.	Kidney	14.80 ± 1.17	15.60 ± 1.67	16.53 ± 4.20
6.	Adrenals	0.39 ± 0.25	0.33 ± 0.05	0.31 ± 0.08
7.	Testes (male)	5.39 ± 2.27	6.11 ± 1.23	3.97 ± 1.45
8.	Uterus (female)	6.489 ± 3.31	7.003 ± 1.42	4.309 ± 2.25
9.	Lungs	9.32 ± 0.99	10.13 ± 0.51	9.46 ± 1.84

Summarized here are the mean (in grams) weights of the various organs removed from the test animals. Animals given the test item showed no discernible differences from those given the vehicle. Calculations were done using a mean and standard deviation format, and a significance level of $p<0.05$ was reached. Group 1 received no treatment (sham group); Group 2 received En-hLMSCs; and Group 3 received En+ hLMSCs. Statistical analysis was done with t-test.
